# Sonication – further progress in the microbiological diagnosis in implant-associated infections

**DOI:** 10.1186/1471-2334-14-S7-O35

**Published:** 2014-10-15

**Authors:** Daniela Tălăpan, Raluca Mihăilescu, Olga Mihaela Dorobăț, Vlad Predescu, Rodica Marinescu, Olivera Lupescu, Florian Purghel, Marius Niculescu, Răzvan Ene, Alexandru Hera, Daniela Munteanu, Mariana Constantin, Emilia Căpraru, Angelica Tenita, Dana Jianu, Oana Săndulescu, Anca Streinu-Cercel, Monica Cârstoiu, Cătălin Cârstoiu, Victoria Aramă, Adrian Streinu-Cercel, Alexandru Rafila

**Affiliations:** 1 National Institute for Infectious Diseases “Prof. Dr. Matei Balş”, Bucharest, Romania; 2 Carol Davila University of Medicine and Pharmacy, Bucharest, Romania; 3 Emergency Clinical Hospital “Sf. Pantelimon”, Bucharest, Romania; 4 Clinical Hospital Colentina, Bucharest; 5 Emergency Clinical Hospital, Bucharest; 6 Clinical Emergency Hospital “Prof. Dr. Bagdasar Arseni”, Bucharest, Romania; 7 Proestetica Medical Center, Bucharest, Romania; 8 University Emergency Hospital of Bucharest, Romania

## Background

Although recommended, sonication is not yet a routinely used technique in the diagnosis of implant-related infections worldwide. So far, this laboratory technique is performed in Romania in the National Institute for Infectious Diseases “Prof. Dr. Matei Balş” since 2012.

## Methods

We evaluated the medical implants received between July 2012 and July 2014. The explanted implants were sonicated using BactoSonic ultrasonic bath (Bandelin, Germany) and the resulting sonication fluid was cultured according to the Trampuz method (NEJM 2007). Microbial identification and antibiotic susceptibility testing was performed using Vitek 2 Compact (BioMerieux, France).

## Results

We sonicated 58 medical implants as follows: 39 orthopedic implants (21 hip prostheses, 11 knee prostheses and 7 fixation devices), 9 breast implants and 10 other devices (e.g. central venous catheter, drainage tube).

We identified a good correlation (93.75%) between sonication culture and clinical findings for orthopedic implants, 22 infections being monomicrobial and 8 polymicrobial. Almost 2/3 of the monomicrobial infections were caused by Gram-positive aerobic cocci: 7 coagulase-negative methicillin-resistant staphylococci (4 *Staphylococcus epidermidis*, 2 *Staphylococcus warneri*, 1 *Staphylococcus haemolyticus*), 7 *Staphylococcus aureus* (5 MRSA and 2 MSSA) and 1 *Streptococcus agalactiae*.

Monomicrobial infections caused by Gram-negative bacilli were as follows: 4 with non-fermenting rods (2 *Burkholderia cepacia*, 1 *Pseudomonas aeruginosa*, 1 *Ochrobactrum anthropi*) and 2 with *Enterobacteriaceae*: *Klebsiella pneumoniae* and *Serratia marcescens*. One infection was caused by Gram-positive anaerobic cocci: *Parvimonas micra*.

Polymicrobial infections were found in 8 cases, 7 with 2 microorganisms and one with 3 (*Klebsiella pneumoniae*, *Pseudomonas aeruginosa* and *Enterococcus faecalis*). Twenty control implants (7 orthopedic, 9 breast and 4 others) were indeed culture negative. Sonication identified infection in 3 additional patients compared to culture of tissue or synovial fluid.

## Conclusion

Easy to perform, sonication improves the microbiological diagnosis by removing bacteria through disruption of the biofilm on explanted prosthetic material revealing both monomicrobial and polymicrobial infections. Anaerobic infections that might be under-detected using conventional methods may be revealed using this technique. Sonication is applicable in multiple medical and surgical specialties (Orthopedics, Neurosurgery, Cardiology, Nephrology, etc.).

